# Humoral immune factors and asthma among American Indian children: a case–control study

**DOI:** 10.1186/s12890-016-0257-6

**Published:** 2016-06-13

**Authors:** Lyle G. Best, Rae A. O’Leary, Marcia A. O’Leary, Joseph M. Yracheta

**Affiliations:** Missouri Breaks Industries Research Inc, Eagle Butte, SD USA; Turtle Mountain Community College, Belcourt, ND USA; 1935 118th Ave NW, Watford City, ND 58854 USA

**Keywords:** Asthma, Airborne allergens, Immunoglobulin, IgE, Allergen specific IgE, C-reactive protein, Eosinophils, American Indian, Children

## Abstract

**Background:**

Asthma is recognized as intimately related to immunologic factors and inflammation, although there are likely multiple phenotypes and pathophysiologic pathways. Biomarkers of inflammation may shed light on causal factors and have potential clinical utility. Individual and population genetic factors are correlated with risk for asthma and improved understanding of these contributions could improve treatment and prevention of this serious condition.

**Methods:**

A population-based sample of 108 children with clinically defined asthma and 216 control children were recruited from a small community in the northern plains of the United States. A complete blood count, high sensitivity C-reactive protein, total IgE and specific antibodies to 5 common airborne antigens (CAA), in addition to basic demographic and anthropomorphic data were obtained. Logistic regression was primarily used to determine the association between these humoral factors and risk of asthma.

**Results:**

The body mass index (BMI) of those with asthma and their total leukocyte counts, percentage of eosinophils, and levels of total IgE were all greater than corresponding control values in univariate analysis. The presence of detectable, specific IgE antibodies to five common airborne antigens was more likely among cases compared with controls. In multivariate analysis, total IgE was independently associated with asthma; but not after inclusion of a cumulative measure of specific IgE sensitization.

**Conclusion:**

Many previously reported associations between anthropomorphic and immune factors and increased risk of asthma appear to be also present in this American Indian population. In this community, asthma is strongly associated with sensitization to CAA.

## Background

Asthma has long been recognized as intimately related to immunologic factors and inflammation [1]. Clinically there appear to be a number of asthma “phenotypes”, with those involving inflammation and immune response ranging from very acute, specific reactions to animal dander for example, to more subtle, low-level degrees of chronic inflammation [2]. To inform therapies, there has been a concerted effort to discover and evaluate various biomarkers of inflammation in general, as well as innate and specific, adaptive immunity [3]. Initial attempts to characterize inflammation showed that crude measures, such as neutrophil count [4] and proportion of eosinophils [5] are associated with asthma. Later, the measurement of total immunoglobulin E (IgE) [6] and then specific immunoglobulins targeting particular exposures, such as certain insect and rodent species was found to be informative [7]. Most recently very detailed and specific components of the inflammatory and immune response are being explored [3].

The complex interplay between the immune system and environmental exposures precludes any simplistic models of asthma pathophysiology; and makes inferences from epidemiologic data difficult. Thus the differing ecological and socio-economic environment of populations can produce characteristic profiles of various immune and inflammatory measures [8]. The “hygiene theory” of asthma etiology is predicated on this basis [9]; and the environmental impact of the microbiome will also require consideration in the future [10].

Lastly, the population genetics of a community sets the stage for this complex interaction between physiology and environment. Differences in immune response and inflammatory status have been seen in comparisons between ethnic groups [11, 12] and more specifically influenced by certain genetic variants [13, 14].

For all of the above reasons, we sought to obtain additional information from an American Indian population, comparing humoral immune factors among those with and without asthma.

## Methods

The measures reported here derive from a case–control study of the environmental and genetic influences on risk of asthma among an American Indian population in north-central United States. Most primary medical care for this community of predominantly tribal members is provided mainly by federal funding to the Indian Health Service (IHS) and a tribal health department. Specialty pulmonology or allergy medical care is a minimum of 160 miles from the demographic center of the community.

This community is located in the north central portion of South Dakota in an area covering 4266 square miles, which is larger than the state of Connecticut. The area’s population is about 8500, resulting in a population density of between 2 and 3 people per square mile. Most live in cluster housing near small towns or in cluster sites far removed from basic services. Beyond federally supported work in health care and education, ranching and farming provide the bulk of employment. None the less, 33 and 42 % of two counties within this community have incomes below the poverty line, making them the 11^th^ and 4^th^ lowest per capita income counties in America respectively. In addition, over 20 % have less than a high school education [15].

Cases were ascertained through automated query of the IHS electronic medical records system, searching for an inclusive array of ICD-9 codes between 493.00 and 493.92, in addition to codes 786.07 (wheezing) and V17.5 (family history of asthma). The search was limited to individuals between and including the ages of 6 and 17 years. Additional cases were sought by contact with local non-IHS providers. This identified over 700 individuals, who were then consented for further review of medical records to determine potential eligibility.

Case definition criteria required at least 2 of the following 3 criteria:

1. a diagnosis of asthma on at least 2 occasions by more than one provider during the past 2 years.
2. refills of asthma treatment medications on at least 2 occasions during the past 2 years.
3. If spirometry had been conducted, there was an improvement in forced expired volume at one second (FEV1) of 20 % with use of albuterol metered-dose inhaler (MDI) or nebulizer

Exclusionary criteria were:

1. Birthweight less than 2500 g
2. Neonatal ventilator treatment
3. Hospitalization at birth greater than 15 days.
4. Congenital heart anomaly requiring surgery
5. Diagnosis of cystic fibrosis
6. Congenital lung, diaphragm, chest wall, or airway anomaly
7. Diagnosis of pneumonia, pertussis, or tuberculosis within the past year
8. Congenital muscular disorder

It was found that many of the potential cases initially identified had been assigned an ICD-9 code indicating “asthma” by the pharmacist filling a prescription for a bronchodilator, although the prescribing physician had not indicated a diagnosis of typical “asthma” and was intending to ameliorate the bronchospastic component of a pulmonary infection. These latter children did not meet diagnostic criteria; but did require considerable recruiter effort to contact parents and determine their status via medical record review.

For each case, two controls were initially recruited by identifying the two children born the day after and before the index case and contacting the parents for consent to review medical records for possible inclusion. As the study progressed, this methodology proved too time consuming and many controls were later recruited from previously identified families with children born almost exclusively (>99 %) within 6 months of the index case. Initial recruitment was concentrated on cases and later focused on controls, thus the inherent bias of controls being older at the time of exam (even though birth dates were generally within protocol limits) was increased, such that all but 5 (2.3 %) of the pairs were examined within one year of each other. Controls met the same exclusionary criteria as cases in addition to:

1. No diagnosis of asthma by any provider during the past 2 years.
2. No prescriptions of any asthma meds during the past 2 years.
3. If spirometry had been conducted, an FEV1 greater than 80 % of the predicted value [16]

Consenting cases and controls were then examined according to study protocol, which included anthropomorphic measures, spirometry, salivary DNA collection and a non-fasting blood draw. Environmental measures of home air quality and dust exposure were made.

The inflammatory and immune biomarkers reported here are as follows:

1. complete blood count (CBC) done at the local IHS laboratory using Abbott Laboratories dedicated reagents on an Abbott CELL-DYN Ruby, Abbott Laboratories, Abbott Park, IL. These were accomplished within 8 h of drawing.
2. high sensitivity C-reactive protein (CRP) done at Sanford Laboratories, Rapid City, SD, using a particle enhanced immunoturbidimetric assay, (Roche dedicated CRPHS reagent, catalog number 04628918190, Roche Diagnostics, Indianapolis, IN), on a Roche Cobas 6000 instrument.
3. total IgE was done at Sanford Laboratories, Sioux Falls, SD by Fluorescent Enzyme Immunoassay, (ImmunoCAP dedicated reagents, catalog number 10-9319-01, Phadia AB, Uppsala, Sweden) on the Phadia ImmunoCAP 250 instrument.
4. allergen specific IgE sensitive to dog and cat dander, A alternata mold, D farinae mite, and B germanica cockroach antigens. These measures were also conducted at Sanford Laboratories, Sioux Falls, SD by the same technique as the total IgE, using ImmunoCAP dedicated reagents, catalog numbers 14-4110-01, 14-4109-01, 14-4106-01, 14-4108-01, 14-4224-01 respectively, Phadia AB, Uppsala, Sweden).

A questionnaire collected social, demographic, and medical history from both cases and controls, most of which will be reported in the future. One question: “Has a medical person ever said that your child had hay fever or seasonal allergies?” sought to gauge atopic symptomatology.

With the exception of age, the majority of variables measured had non-normal distributions (e.g. skewedness >1) and were natural log transformed prior to hypothesis testing. All of the resulting distributions, except those representing a quantitative value for specific IgE sensitization, exhibited a skewedness of less than 0.685 after transformation. The distribution of specific IgE values remain significantly skewed, even after log-transformation (maximum skewedness of 3.6 for Alternaria), probably due to the large proportion of measures below the detection limit. Descriptive statistics report median, minima and maxima, and 95 % confidence intervals for non-normal variables. Cases and controls were compared using McNemar chi-square statistics for discrete variables and paired *t*-test for continuous variables. Univariate and multivariate logistic regression was used to explore effects of multiple biomarkers on risk of asthma. Specific IgE levels were analyzed both as a dichotomized variable (above or below the detection threshold) and as a continuous variable, with those below the detection limit (0.35 kUA/L) assigned a value of 0.35 divided by the √2, as is commonly done [17]. All models were adjusted for age, as there was a significant difference between cases and controls due to factors noted previously. Correlation results are expressed as Pearson coefficients, rho (ρ). Descriptive analyses were done using SPSS 13.0.1 (IBM Inc, Armonk, NY). Logistic regression used LogXact-11 (Cytel Inc, Cambridge, MA). Significance was considered demonstrated at *p* < 0.05.

## Results

The characteristics of cases and controls are shown and compared in Table 1.

**Table 1:** Characteristics of cases and controls. Represented as count and percent of subgroup (N, %) or as median and interquartile range (IQR). P value determined McNemar chi-squared test, or paired samples *t* test using natural log transformed measures

	Cases		Controls		
	*N* = 108		*N* = 216		
Characteristic	Median or N (%)	Min - Max	Median or N (%)	Min - Max	*P* value
		95 % CI (mean)		95 % CI (mean)	
Male, N (%)	57 (53 %)	43.4–63.2	109 (51 %)	57.1–43.8	0.634
Age (years)	11.81	6.1–17.9	12.12	6.1–18.3	0.001
		11.4–12.2		11.7–12.6	
BMI (kg/m2)	24.4	14.6–62.1	21.9	10.8–45.4	0.004
		24.3–26.5		22.7–24.4	
White Blood Cells (WBC) (X 1000)	7.30	3.3–16.5	6.80	2.7–14.7	0.001
		7.2–7.9		6.7–7.1	
PMNs^a^ (% of WBC)	54.7	25.7–85.1	56.2	3.6–85.5	0.398
		53.4–56.1		54.7–57.5	
Lymphocytes (%)	30.9	9.0–65.6	30.8	12.0–54.8	0.797
		30.4–32.8		30.2–35.5	
Basophils (%)	1.3	0.5–6.3	1.5	0.3–17.0	0.273
		1.4–1.6		1.5–1.9	
Eosinophils (%)	4.1	0.3–22.3	2.7	0.1–27.2	0.002
		4.8–5.6		3.3–4.2	
CRP (mg/L)	1.12	0.35–17.2	1.07	0.36–74.4	0.254
		2.0–2.8		1.4–3.5	
IgE^b^ (total, kU/L)	213	3.0–4473	89	2.0–2676	0.001
		382.6–573.9		169.1–271.0	
IgE, Alternaria^c^	24 (22 %)	14.4–30.1	11 (5 %)	2.2–8.0	0.001
IgE, cat dander	34 (32 %)	22.7–40.2	17 (8 %)	4.3–11.5	0.001
IgE, cockroach	29 (27 %)	18.5–35.2	28 (13 %)	8.5–17.4	0.001
IgE, dust mite	40 (37 %)	27.9–46.1	39 (18 %)	12.9–23.2	0.001
IgE, dog dander	30 (28 %)	19.3–36.2	13 (6 %)	2.8–9.2	0.001
> one antigen over detection limit	66 (61 %)	51.9–70.3	61 (28 %)	22.2–34.2	0.001
History of hay fever or seasonal allergy	38 (36 %)	26.2–44.2	65 (32 %)	24.0–36.2	0.444

^a^polymorphonuclear leukocytes

^b^total Immunoglobulin E

^c^antigen specific IgE (ie above minimum detection limit, <0.35 kUA/L)

The significant difference in age between cases and controls is probably due to a number of factors, as noted in the methods section. The mean difference in age is approximately 4 months; but for this reason all logistic regression models were adjusted for age.

Notable differences between case and control groups include body mass index (BMI), white blood cell count (WBC), percent eosinophils, total IgE, and specific IgE levels over the detectable limit for all tested antigens. Among controls the geometric mean total IgE was 88.23 kIU/L and the median was 89 kIU/L, with an interquartile range of 35.5 to 229. There was no significant statistical association between a history of hayfever or seasonal allergy and either atopy (as defined by at least one of the five specific IgE assays) or total IgE. Among cases, 61 % had at least one specific IgE above the detectable limit, compared with only 28 % of controls.

Table 2 shows the results of univariate logistic regression models testing the association of various measures with case/control status. Relationships between case/control status and covariates were similar to those seen in Table 1, except that age did not reach statistical significance in this analysis. The specific IgE levels were analyzed both as continuous and binary variables (more or less than the detectable limit). All of the tested specific allergens showed very significant association with asthma in both binary analysis and quantitative analyses. An ordinal variable comprised of the cumulative number of all of the five antigens above the detectable limit was highly associated with case status (*p* = 0.001).

**Table 2:** Logistic regression analysis with adjustment for age due to significant age difference between cases and controls. Odds ratios indicate increased risk associated with a 1 unit change in the natural log of continuous variables

	Odds Ratio	*P*-Value	95 % CI
Age (years)	0.968	0.377	0.90–1.04
Individual variables below, simultaneously adjusted for age			
Gender (Female = 0, Male = 1)	1.083	0.736	0.68–1.72
Body Mass Index (kg/m^2^)	3.720^a^	0.006	1.46–9.45
CRP (mg/L)	1.143^a^	0.260	0.91–1.44
IgE (total, kU/L)	1.425^a^	0.001	1.20–1.69
White blood cell count (X1,000)	3.008^a^	0.01	1.26–7.20
PMN leukocytes	0.899^a^	0.836	0.33–2.45
Lymphocytes	0.947^a^	0.903	0.39–2.27
Eosinophils	1.520^a^	0.01	1.10–2.09
Basophils	0.753^a^	0.277	0.45–1.26
Alternaria (quant)^b^	1.765 ^a^	0.001	1.34–2.32
Alternaria (binary)^c^	5.280	0.001	2.47–11.27
Cat dander (quant)	1.702^a^	0.001	1.36–2.14
Cat dander (binary)	5.432	0.001	2.86–10.32
Cockroach (quant)	1.612^a^	0.001	1.27–2.05
Cockroach (binary)	2.522	0.002	1.41–4.53
Mite (quant)	1.287^a^	0.02	1.04–1.59
Mite (binary)	2.751	0.001	1.63–4.66
Dog (quant)	2.360^a^	0.001	1.63–3.42
Dog (binary)	6.135	0.001	3.03–12.41
Cumulative sensitivity^d^	1.830	0.001	1.49–2.24

^a^odds ratio associated with the natural log transformed value of this measure

^b^IgE specific titer, samples below the detection limit of 0.35 were assigned values of 0.35 /√2

^c^Any level above detection limit vs below detection limit

^d^Number of antigens tested that showed specific IgE above detection limit for that individual

Results of multivariate logistic regression models are found in Table 3. Covariates with significant association in univariate analysis were entered into two models, with and without the cumulative sensitization variable (CSV). Inclusion of CSV negated the association of total IgE with asthma status. Analysis of total IgE as a binary trait, above and below the median, gave essentially equivalent results.

**Table 3:** Two Multivariate logistic regression analyses with adjustment for age, one including a cumulative measure of specific sensitization and another without

	Odds Ratio	*P*-Value	95 % CI
Age (years)	0.910	0.059	0.83–1.00
Body Mass Index (kg/m^2^)	2.471^a^	0.114	0.81–7.58
IgE (total, kU/L)	0.997^a^	0.981	0.79–1.26
White blood cell count (X1,000)	1.907^a^	0.225	0.67–5.40
Eosinophils (%)	1.054^a^	0.782	0.73–1.53
Cumulative sensitivity^b^	1.751	0.001	1.33–2.30
Multivariate model without cumulative sensitivity			
Age (years)	0.930	0.123	0.85–1.02
Body Mass Index (kg/m^2^)	2.700^a^	0.070	0.92–7.90
IgE (total, kU/L)	1.352^a^	0.001	1.12–1.63
White blood cell count (X1,000)	1.965^a^	0.180	0.73–5.28
Eosinophils (%)	1.158^a^	0.422	0.81–1.66

^a^odds ratio associated with the natural log transformed value of this measure

^b^Number of antigens tested that showed specific IgE above detection limit for that individual

Correlation between certain variables is present. Not surprisingly age is positively correlated with BMI (ρ = 0.423, *p* = 0.001); and negatively correlated with % eosinophils (ρ = −0.310, *p* = 0.001); but notably not correlated with cumulative sensitization (ρ = 0.046, *p* = 0.411). Positive correlations are also seen between total IgE and cumulative BMI, total IgE and CSV (ρ = 0.658, *p* = 0.001), BMI and WBC (ρ = 0.349, *p* = 0.001); and total IgE and % eosinophils (ρ = 0.392, *p* = 0.001).

## Discussion

Asthma is a multifactorial condition with significant associated morbidity, mortality and public health consequences for all populations [2]; but American Indian communities in particular, due to geographic isolation and the paucity of services [18–20]. Although asthma results in differing clinical presentations, historically, the importance of immune factors has been recognized as playing a key role [21]. A clearer understanding of the immune factors influencing asthma offers the possibility of further improvements in prevention, diagnosis and control, beyond those seen in the latter from the increased use of inhaled corticosteroid medications in recent decades [22].

The findings of this case–control study among 324 American Indian children in the Northern Plains of the United States are largely in accord with previous evidence of increased biomarkers of inflammation and specific, immune sensitization. We demonstrated increased total IgE immunoglobulins among those with asthma (although not in a multivariate model including a measure of specific sensitization), as also reported in studies of European [12, 23–25], Han Chinese [26], African Americans and various Hispanic populations [11, 27]. Other studies have been unable to find an association between total IgE and asthma [28] or found this association holds only among those with atopic sensitization to specific antigens [29]. There is debate as to whether the increased IgE is in the pathophysiologic pathway of asthma, or merely an associated “epiphenomenon” [30, 31]; however this cannot be answered by a cross-sectional study such as the present. Differences in the activity of dendritic cells and IgA response likely play a modulating role in the immune effects of IgE and other downstream contributors [32]. Lack of strong evidence relating asthma and genetic factors that increase IgE levels is also an argument against a mechanistic role for total IgE [6, 30]; none the less, anti-IgE therapy appears to ameliorate the symptoms of asthma [33].

The level of high sensitivity CRP did not show any association with case–control status. This finding is unexpected in light of various reports in the literature [34, 35], although one report found elevated CRP levels among infants protective for subsequent asthma, in accord with the “hygiene hypothesis” [36].

The present study also documents increased levels of all leukocytes and specifically the proportion of eosinophils among those with asthma. However, these measures are not found to be independent factors in models adjusting for total IgE or a cumulative measure of IgE antibodies targeting putative asthma triggers. Whether this represents different aspects of a common inflammatory factor, or a more direct influence of IgE on eosinophils (or vice versa) is not clear. The literature has focused primarily on the presence of total leukocytes and eosinophils in bronchial washings; but there are reports of increased numbers of both elements in peripheral circulation as well [4, 37–39].

In the present study, IgE antibodies specific for the five common environmental antigens tested are strongly associated with asthma. This is apparent for each of the five antigens when analyzed as a binary variable (above or below the limit of detection); and not surprisingly, when a cumulative variable incorporating all of the antigens was used. The increased risk of asthma in the presence of specific IgE antibodies is commonly reported in the literature [25] and is frequently included in diagnostic criteria for atopy [31] and “extrinsic” asthma [40]. Of some interest is the comparison between reported prevalence of sensitization (at least one of 5 antigens above the detection limit) in the present study (28 % among controls) with controls from an Alaskan Native [28] (18 %), a Swedish [41] study (3 %), and a US NHANES, population-based sample (37 %) [29].

Although obviously not a humoral factor, univariate analyses showed obesity was also positively associated with asthma risk in this cohort, with each BMI unit contributing approximately 5 % additional risk. The contribution of BMI was attenuated in multivariate analyses; but was still suggestive (*p* = 0.07) when a cumulative measure of sensitization was excluded from the model. The increased risk of asthma in the presence of obesity is well established in the literature [42, 43]; and has been previously demonstrated in an American Indian community [44]. This appears to exemplify still one more aspect of the complicated relationship between obesity and inflammation [45].

While there are clearly numerous social and environmental factors that influence the risk of asthma and its complications, there also appear to be ethnic and genetic influences at play [46, 47]. Due to differences in study populations, comparisons invite caution; but the total IgE geometric mean (GM) among controls in the present study (89 kU/L), is above the GM for European and African American controls in Michigan [12] (24.8 and 59.2 kU/L respectively), median non-atopic Greenlandic children (61 kU/L) [48], similar to a Japanese cohort (mean 81.3 kU/L) [49] and below the GM of an Alaskan Native cohort (122.1 kU/L) [28]. In addition, 45.5 % of total IgE levels among controls are above the reference range (20–100 kU/L) for the study lab. It has been hypothesized that population-level regulation of IgE may reflect historical, environmental exposures, for example to parasitic infestation [50].

## Conclusion

Evidence from this case–control study among an American Indian population shows strong, independent association between increasing levels of specific IgE sensitization to 5 common environmental antigens and asthma, even after adjustment for other widely accepted risk factors, such as obesity and total IgE levels. The presence of elevated total IgE levels is associated with asthma risk as expected, when there is no adjustment for specific IgE sensitization. In light of evidence that asthma risk is influenced by genetic variants, the current results complement our understanding of asthma, especially as it relates to ethnicity.

## Abbreviations

BMI, body mass index; CAA, common airborne antigens; CRP, C-reactive protein; CSV, cumulative sensitization variable; FEV1, forced expired volume at one second; GM, geometric mean; ICD-9, International Classification of Diseases, ninth edition; IgE, total immunoglobulin E; MDI, metered-dose inhaler; WBC, white blood cell
